# An Assessment of Temporal and Spatial Dynamics of Regional Water Resources Security in the DPSIR Framework in Jiangxi Province, China

**DOI:** 10.3390/ijerph19063650

**Published:** 2022-03-19

**Authors:** Mengtian Lu, Xiaoying Wang, Weihong Liao, Chao Wang, Xiaohui Lei, Hao Wang

**Affiliations:** 1 Institute of Municipal Engineering, College of Civil Engineering and Architecture, Zhejiang University, Hangzhou 310030, China; 11512052@zju.edu.cn (M.L.); wanghao@iwhr.com (H.W.); 2 Anhui Water Conservancy Technical College, Hefei 231603, China; wxy769662707@163.com; 3 Key Laboratory of Simulation and Regulation of Water Cycle in River Basin, China Institute of Water Resources and Hydropower Research, Beijing 100038, China; wangchao@iwhr.com (C.W.); lxh@iwhr.com (X.L.)

**Keywords:** water resources security, DPSIR, confidence threshold method

## Abstract

Water resources are critical for the survival and prosperity of both natural and socioeconomic systems. A good and informational water resources evaluation system is substantial in monitoring and maintaining sustainable use of water. The Driver-Pressure-State-Impact-Response (DPSIR) framework is a widely used general framework that enabled the measurement of water resources security in five different environmental and socioeconomic subsystems: driver, pressure, state, impact, and response. Methodologically, outcomes of water resources evaluation based on such framework and using fuzzy set pair analysis method and confidence interval rating method depend critically on a confidence threshold parameter which was often subjectively chosen in previous studies. In this work, we demonstrated that the subjectivity in the choice of this critical parameter can lead to contradicting conclusions about water resources security, and we addressed this caveat of subjectivity by proposing a simple modification in which we sample a range of thresholds and pool them to make more objective evaluations. We applied our modified method and used DPSIR framework to evaluate the regional water resource security in Jiangxi Province, China. The spatial-temporal analysis of water resources security level was carried out in the study area, despite the improvement in Pressure, Impact, and Response factors, the Driver factor is found to become less safe over the years. Significant variation of water security across cities are found notably in Pressure and Response factors. Furthermore, we assessed both cross-sectionally and longitudinally the inter-correlations among the DPSIR nodes in the DPSIR framework. The region-specific associations among the DPSIR nodes showed important deviances from the general DPSIR framework, and our analysis showed that in our study region, although Responses of regional government work effectively in improving Pressure and State security, more attention should be paid to improving Driver security in future regional water resources planning and management in Jiangxi Province, China.

## 1. Introduction

Water resources are the basis of human survival and development and are irreplaceable natural resources for sustainable economic and social development [1]. Since the 1970s, the rapid growth of world population and the rapid development of the global economy have led to the rapid growth of global water consumption and water pollution [2,3]. In recent years, under the influence of global climate change and high-intensity human activities, the water cycle and the spatial and temporal distribution of water resources have undergone complicated changes. The complexity of hydrological characteristics and the insecurity of water resources increased substantially [4,5]. Therefore, water resources security evaluation and the selection of appropriate evaluation methods is of critical importance in monitoring the sustainable use of water resources and guiding countries and regions to maintain socially sustainable development [6,7].

To evaluate water resources security, scholars have come up with various indicators to measure the degree of regional water resources security, such as per capita water resources and water resources vulnerability index [8,9]. Measuring water resources security via per capita water resources is proposed by Falkenmark et al. [10]. The vulnerability index of water resources refers to the percentage of annual freshwater resources taken up in the total amount of available or renewable freshwater resources. Raskin et al. [11] used a vulnerability index and classified water resource pressure as low, medium low, medium high, and high based on the degree of water resources usage. Other commonly used water resources security evaluation indicators include the water resources development and utilization index [12], the water allocation and priority strategy index [13], and water poverty index [14].

Recently, more and more studies evaluated water resources security from a multi-dimensional perspective that utilizes a system of indicators from different domains [15,16,17,18,19,20]. Various multi-dimensional water resources evaluation frameworks and methods have been developed, including methods based on catastrophe theory [15], system dynamics model (SDM) [16,17], process analysis method (PAM) [21], WaterGAP3 modeling framework [18], projection pursuit model [19], and multistage integrated method [20]. For a comprehensive review of water resources evaluation tools, see [22]. Among them, one of the most commonly used frameworks is the Driver-Pressure-State-Impact-Response (DPSIR) framework. Compared to other frameworks like SDM and PAM, the DPSIR framework includes more measures and is more flexible [23]. The DPSIR framework has been widely applied in water resources and ecological security assessment studies [19,24,25,26,27,28,29,30,31,32,33,34,35,36,37,38,39,40,41,42,43,44]. Some primitive versions and new variants of the DPSIR framework were also used in the literature, e.g., PSR model [45,46], DPSI model [33], PSIR model [47,48], DPSR model [49], and DPSIRM model [36,50].

The DPSIR model was proposed by the European Environmental Agency in 1995 [51,52] and has been widely used in policymaking and research. The DPSIR model has the advantage of linking among several components in the water resources security assessment system, and it allows for analyzing the coupling relationship between natural environment resources and human activities. The DPSIR model aims to establish a causal chain of Driver-Pressure-State-Impact-Response, and these five different sub-systems have different implications [24,27,30,33,53]. “Driver” refers to the socio-economic or socio-cultural factors that promote the increase or decrease of water system pressure. The Driver sub-system includes factors like population growth, prosperity level, social or technological change, etc. “Pressures” is mainly reflected by the direct pressure of human behavior on natural resources and the environment. The Pressures sub-system includes factors like water usage and wastewater discharge. “State” is the condition of the environment under various pressure factors. The State sub-system includes factors like water resource quantity and quality. “Impact” refers to the consequences of environmental conditions, which represents the observable positive or negative results, such as human health impact or vegetation damage. “Response” indicates the countermeasures taken by mankind in the process of promoting sustainable development, such as improving resource utilization efficiency, reducing pollution, increasing investment, etc. In summary, the DPSIR model evaluates threats from social, economic, and human activities to regional water resources security and the human responses to these threats.

China is a country with serious uneven spatial and temporal distribution of water resources, and water resources problems are very prominent. Water resources shortage, drought, and flood disasters and water ecological environment problems have become important factors restricting China’s economic development. Water resources security evaluation has received significant interests among scholars [7,16,20,27,30,36,45,54,55,56,57,58,59,60]. Regional water resources security evaluation has been done at both river basins [37,43,44,61,62,63] and urban areas which are analyzed both at the level of individual cities [15,41,45,46,58,64,65] and at the level of provinces [16,20,27,47,54,56,65,66,67,68]. Compared to other provinces in China, Jiangxi is a province relatively rich in water resources [65]. However, the temporal and spatial distribution of water resources in Jiangxi is uneven, and seasonal water resources are scarce. With the rapid development of the economy and the acceleration of urbanization, the contradiction between supply and demand of water resources is becoming increasingly prominent, the quality of water environment is declining year by year, and water pollution emergencies occur from time to time [66]. How to reasonably develop water resources and achieve sustainable utilization is an important and arduous task.

In this paper, we evaluated the regional water resources security in Jiangxi Province, China, using the DPSIR framework. The study period was chosen based on the availability of data in Jiangxi Province, China. The entropy weight method [69] was used to calculate the indices weight, and the fuzzy set pair analysis method [70] was used to evaluate the water resources security. Instead of using a subjective confidence threshold parameter to draw boundaries between Safe vs. Unsafe, which has been used by many studies in the literature [38,39,40,41], we demonstrated that the limitation of such method is that subjectivity in the choice of the confidence threshold could lead to contradictory conclusions. Furthermore, we addressed this issue of subjectivity by proposing a modified method that samples a range of thresholds to obtain a more objective measure of water resources security. We evaluated the temporal and spatial dynamics of water resources security in the Driver force, Pressure, State, Impact, and Responses domains in 11 cities in Jiangxi Province over the period of 2010–2018 using our modified method. In addition, we empirically assessed the inter-correlations among the DPSIR nodes over time and space using repeated measures correlation. Our analysis revealed a more complicated and region-specific flows of interactions in the DPSIR framework. Our approach estimates region-specific sensitivities and associations among the DPSIR sub-systems and can use this information to better guide local policy makers on improving the carrying capacity of water resources and strengthening the sustainable development of economy, society, and water resources in the Jiangxi Province.

## 2. Study Area and Datasets

### 2.1. Study Area

**Figure 1:**
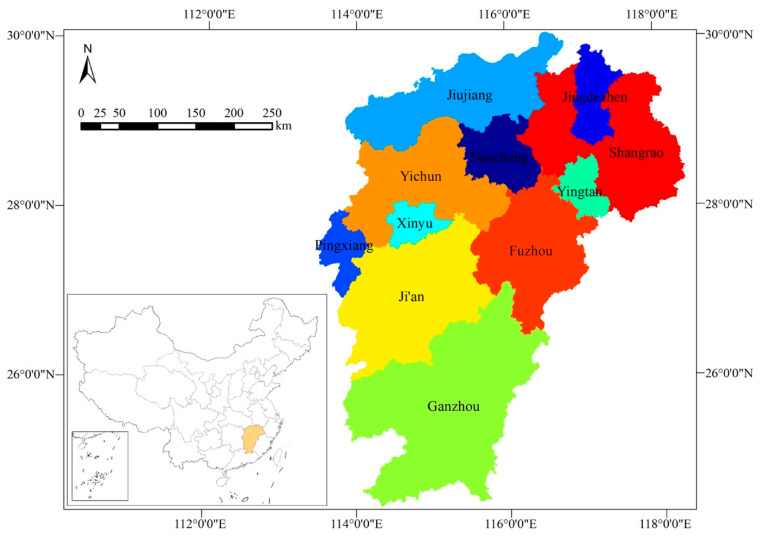
Study area: Jiangxi Province, China.

The Jiangxi Province (113°34′36″–118°28′58″ E, 24°29′14″–30°04′41″ N) lies in the southern bank of the middle and lower reaches of the Yangtze River and is one of the most important inland provinces in China. It belongs to East China, with a land area of 166,900 square kilometers, accounting for 1.8% of the national land area, with a total population of more than 46.47 million and jurisdiction over 11 prefecture level cities, namely, Nanchang, Jingdezhen, Pingxiang, Jiujiang, Xinyu, Yingtan, Ganzhou, Ji’an, Yichun, Fuzhou, and Shangrao. The geographical locations of the cities are shown in Figure 1. In this paper, we will evaluate the water resources safety of these 11 cities in Jiangxi Province using the DPSIR framework.

The whole year of Jiangxi has mild climate, with sufficient sunshine, plentiful rainfall, and long frost-free period, which belongs to the subtropical warm and humid monsoon climate. The average annual temperature is about 16.3–19.5 °C. Jiangxi Province is one of the rainy provinces in China, with annual precipitation of 1341–1943 mm. Moreover, 97.7% of the area of Jiangxi belongs to the Yangtze River Basin, with rich water resources and dense river network. There are more than 2400 large and small rivers in Jiangxi Province, with a total length of about 18,400 km. The average annual water resources amount to 156.5 billion cubic meters. The total runoff is the seventh in China, the per capita is the fifth, and the cultivated land is the sixth. The annual average value of groundwater natural resources is more than 21.2 billion cubic meters, accounting for 2.3% of the national natural groundwater resources.

### 2.2. Datasets

The dataset used in this paper to conduct the water resources security evaluation comes from the annual statistical data and official documents approved by regional governmental departments, including China Statistical Yearbook, Jiangxi statistical yearbook, and Jiangxi water resources bulletin. It could be accessed through China’s economic and social big data research platform (https://data.cnki.net/NewHome/index (accessed on 20 November 2020)) and National Bureau of Statistics website (http://data.stats.gov.cn/ (accessed on 20 November 2020)). Given the integrity and availability of data, the study period is chosen to be from 2010 to 2018. Among them, most evaluation index data of driver (D), pressure (P), state (S), influence (I), response (R) can be obtained directly, and some indexes can be obtained by index calculation method.

## 3. Methodology

### 3.1. Water Resources Security Evaluation Index

In this paper, water resources security is evaluated using the DPSIR framework. One domain-specific index is calculated respectively for each of the five subdomains, namely, Driver, Pressure, State, Impact, and Response. These indices are computed for each of the 11 cities and for each year separately. Fifteen social economic factors are used in the calculation of the indices of water resources security in Jiangxi Province. These factors are classified based on the nature of the factor into one of the five subsystems of DPSIR (see Table 1). All the factors used in this paper were well-accepted in the field and were used by multiple studies in the previous literature; the relevant references to each of the 15 factors are listed in the last column of Table 1.

### 3.2. Individual Factor Grade Intervals

Based on the current domestic research results in China and the regional situation of the Jiangxi Province [57], as the first step of the pipeline, for each of the 15 factors (See Table 2), we assign the following 5 security levels: 1—safe, 2—generally safe, 3—barely safe, 4—unsafe, and 5—very unsafe. The specific boundaries used here are chosen based on both standards that have been used in the literature and standards and planning objectives issued by the local government and protection requirements of river water system [58,65,66]. For details, see Table 2, for specific water resources safety classification boundaries.

### 3.3. Fuzzy Security Level Using Fuzzy Set Pair Analysis

To better quantify the dynamics of the DPSIR factors, instead of directly using the classification threshold in Table 2, the water security levels of each of the 5 DPSIR categories are calculated using Fuzzy Set Pair Analysis (FSPA). FSPA is a special case of Set Pair Analysis (SPA) which is a systematic analysis method established by Zhao 1989 [78]. SPA has been applied to various canonical set pairs in the field of hydrology and water resources, e.g., flood vs. drought, qualified vs. unqualified, safety vs. danger [17,70,79,80]. FSPA applies fuzzy logic theory to SPA and accounts for system uncertainty in addition to identity, by considering difference and opposition of two sets of each set pair. In dealing with problems with uncertainty such as evaluating the water security level, instead of using a hard coded boundary to categorize factors into Safe vs. Unsafe, FSPA represents the security level by quantifying the similarity of the current value to that of each security category. FSPA is proved to be effective and has been successfully applied to water resources evaluation in many studies [54,56,81,82,83,84,85,86].

#### 3.3.1. Calculation of Index Connection Degree

Assume that the set of water resources security evaluation index system composed of *n* index values $X=\left\{x_{1},x_{2},\cdots ,x_{n}\right\},\;x_{i}\in \left\{1,2,3,\ldots ,K\right\}$ . K is the number of the evaluation grade standard, in our case *K* = 5 since we have 5 different water security levels. The evaluation grade standard is $B_{k}\;(k=1,2,\cdots ,K)$ , and here, the set of the $k^{th}$ level of the evaluation grade standard is $B_{k}=\left\{k,k,\ldots ,k\right\}$ . The similarity between the two sets *X* and $B_{k}$ represents how close *X* is to a security level of *k*.

Here, we define the index connection degree between *X* and $B_{k}$ , for each *k*. First, we compute the difference between each element of *X* and $B_{k}$ , $\left|x_{i}-k\right|$ . Then, we count the number of occurrences that $|x_{i}-k|=i$ , and denote it as $F_{i}$ . In particular $F_{0}$ is the number of times that $x_{i}=k$ , and $F_{k-1}$ is the number of times that $\left\{x_{i},\;k\right\}=\left\{1,k\right\}$ . Then the *K*-element connection degree of $\left(X,B_{k}\right)$ is

(1) $$\mu_{X∼B_{k}}=b_{0}I_{0}+b_{1}I_{1}+b_{2}I_{2}+\cdots +b_{K-2}I_{K-2}+b_{K-1}I_{K-1}$$

where $b_{i}=\frac{F_{i}}{n}$ and $b_{0}+b_{1}+b_{2}+\cdots +b_{K-2}+b_{K-1}=1$

$I_{1},\;I_{2},\;\ldots ,\;I_{k-1}$ are the coefficients of difference degree component, which can be determined by uniform value method e.g., $I_{i}=1-\frac{2i}{K-1}$ ; $I_{K-1}$ is the coefficient of contrary degree which is usually taken as −1.

In this paper, $B_{k}$ is specified as the set $B_{1}$ composed of the first level evaluation criteria of a certain index. In case of $\left(X,\;B_{1}\right)$ , $b_{0}=\frac{F_{0}}{n}$ is the identical degree of $\left(X,B_{k}\right)$ which represents the possibility of index X belonging to the $1^{st}$ level of the standard; $b_{1}=\frac{F_{1}}{n}$ , $b_{2}=$ $\frac{F_{2}}{n}$ , $b_{K-2}=$ $\frac{F_{K-2}}{n}$ are the difference degree which represents the possibility of index *X* belonging to the $2^{nd}$, $3^{rd},$ and the (K−1) level of the standard; $b_{K-1}=\frac{F_{K-1}}{n}$ is the contrary degree which represents the possibility of index $x_{t}$ belonging to the *K^th^* level.

Because the boundary of grade standard is fuzzy, the degree of connection $\mu_{X_{t}∼B_{1}}$ can be calculated using Equations (2) and (3). $\;X_{t}$ is the factor of interest (D1–D4, P1–P4, S1–S2, I1–I3, R1–R2) at year *t*. $s_{1},\;s_{2},\;\ldots ,\;s_{K-1}$ are the grade boundaries for factor *X_t_* as indicated in Table 2.

Generally, the indicators of water resources security evaluation can be divided into cost indicators (negative indicators) and benefit indicators (positive indicators). For the cost indicators (the smaller the better), when K>2 , the *K*-element connection degree of $\left(X_{t},B_{1}\right)$ is

(2) $$\mu_{X_{t}\sim B_{1}}=\{\begin{array}{c} 1I_{0}+0I_{1}+0I_{2}+\;\cdots \;+0I_{K-2}+0I_{K-1},\;\;\;\;\;\;\;\;\;\;\;\;\;\;\;x_{t}\leq s_{1};\\ \frac{s_{1}+s_{2}-2x_{t}}{s_{2}-s_{1}}I_{0}+\frac{2x_{t}-2s_{1}}{s_{2}-s_{1}}I_{1}+0I_{2}+\;\cdots \;+0I_{K-2}+0I_{K-1},\;\;\;\;\;s_{1}<x_{t}\leq \frac{s_{1}+s_{2}}{2};\\ 0I_{0}+\frac{s_{2}+s_{3}-2x_{t}}{s_{3}-s_{1}}I_{1}+\frac{2x_{t}-s_{1}-s_{2}}{s_{3}-s_{1}}I_{2}+\;\cdots \;+0I_{K-2}+0I_{K-1},\;\;\;\;\;\frac{s_{1}+s_{2}}{2}<x_{t}\leq \frac{s_{2}+s_{3}}{2};\\ \begin{array}{c} \cdots \\ \begin{array}{c} 0I_{0}+0I_{1}+\;\cdots \;+\frac{2s_{K-1}-2x_{t}}{s_{K-1}-s_{K-2}}I_{K-2}+\frac{2x_{t}-s_{K-1}-s_{K-2}}{s_{K-1}-s_{K-2}}I_{K-1},\;\;\;\;\;\;\;\;\;\;\;\;\;\;\;\frac{s_{K-2}+s_{K-1}}{2}<x_{t}\leq s_{K-1};\\ 0I_{0}+0I_{1}+0I_{2}+\;\cdots +0I_{K-2}+1I_{K-1},\;\;\;\;\;\;\;\;\;\;\;\;\;\;\;\;\;x_{t}>s_{K-1} \end{array} \end{array} \end{array}$$

where $s_{1}\leq s_{2}\leq \cdots \leq s_{K-1}$ .

For the benefit indicators (the bigger the better), when K>2 , the *K*-element connection degree of $\left(X_{t},B_{1}\right)$ is

(3) $$\begin{array}{c} \mu_{X_{t}∼B_{1}}=\{\begin{array}{c} 1I_{0}+0I_{1}+0I_{2}+\;\cdots \;+0I_{K-2}+0I_{K-1},\;\;\;\;\;\;\;\;\;\;\;\;\;\;\;x_{t}\geq s_{1};\\ \frac{2x_{t}-s_{1}-s_{2}}{s_{1}-s_{2}}I_{0}+\frac{2s_{1}-2x_{t}}{s_{1}-s_{2}}I_{1}+0I_{2}+\;\cdots \;+0I_{K-2}+0I_{K-1},\;\;\;\;\;\frac{s_{1}+s_{2}}{2}\leq x_{t}<s_{1};\;\\ 0I_{0}+\frac{2x_{t}-s_{2}-s_{3}}{s_{1}-s_{3}}I_{1}+\frac{s_{1}+s_{2}-2x_{t}}{s_{1}-s_{3}}I_{2}+\cdots +0I_{K-2}+0I_{K-1},\;\;\;\;\;\;\frac{s_{2}+s_{3}}{2}\leq x_{t}<\frac{s_{1}+s_{2}}{2};\\ \cdots \\ 0I_{0}+0I_{1}+\;\cdots \;+\frac{2x_{t}-2s_{K-1}}{s_{K-2}-s_{K-1}}I_{K-2}+\frac{s_{K-1}+s_{K-2}-2x_{t}}{s_{K-2}-s_{K-1}}I_{K-1},\;\;\;\;\;\;\;\;\;\;\;\;\;\;\;\;\;s_{K-1}\leq x_{t}<\frac{s_{K-2}+s_{K-1}}{2};\\ 0I_{0}+0I_{1}+0I_{2}+\;\cdots \;+0I_{K-2}+1I_{K-1},\;\;\;\;\;\;\;\;\;\;\;\;\;\;\;\;\;\;x_{t}<s_{K-1} \end{array} \end{array}$$

where $s_{1}\geq s_{2}\geq \cdots \geq s_{K-1}$ .

#### 3.3.2. Determination of Index Weights

After the establishment of the evaluation index system, it is necessary to select an appropriate method to determine the evaluation index weight. At present, the widely used methods include analytic hierarchy process (AHP) [36], expert investigation method, BP neural network technology [87], and entropy weight method [69]. Compared with commonly used alternative methods such as AHP, which involves the construction of a subjective evaluation matrix, the entropy weight method is more objective and can instead objectively weigh the different indices based on implicit information in the index data. The entropy method has been adopted by many previous studies in water resources evaluation [42,44,54,59,60,69]. Thus, in this study, we used the entropy weight method.

Entropy weight method is often used in information theory to calculate index weight. In information theory, information is a measure of the order degree of the system, and entropy represents the disorder degree of the system. When the entropy of an index is smaller, the difference among the index data is more systematic, which indicates a greater influence of the index on the evaluated object, and hence, a greater weight should be assigned. On the contrary, the greater the entropy, the more random the index data, indicating a smaller influence of the index on the evaluated object, and hence, a smaller weight of the index should be assigned. The steps are as follows:

(1) Standardize the evaluation index:

Assume the water resources security evaluation system includes n evaluation objects (for each DPSIR domain, *n* is the number of years of evaluation) and *m* evaluation indexes (for each DPSIR domain, we have m factors). If the $j^{th}$ evaluation index of the $i^{th}$ evaluation object is $x_{ij}\;\left(i=1,2,\cdots ,n;j=1,2,\cdots ,m\right)$ , then the index matrix *X* composed of $x_{ij}$ is as follows:

(4) $$X={\left(x_{ij}\right)}_{n\times m}=\left[\begin{array}{ccc} x_{11} & \cdots & x_{1m}\\ \vdots & \ddots & \vdots \\ x_{n1} & \cdots & x_{nm} \end{array}\right]$$

We compute for the positive index a normalized value:

(5) $$v_{ij}=\frac{x_{i,j}-x_{\min j}}{x_{\max j}-x_{\min j}}$$

We compute for the negative index a normalized value:

(6) $$v_{ij}=\frac{x_{\max j}-x_{ij}}{x_{\max j}-x_{\min j}}$$

Here, $x_{\max}$ is the maximum value of the index, and $x_{\min}$ is the minimum value of the index for column j .

After normalization, the n×m value matrix V is obtained:

(7) $$V={\left(v_{ij}\right)}_{n\times m}=\left[\begin{array}{ccc} v_{11} & \cdots & v_{1m}\\ \vdots & \ddots & \vdots \\ v_{n1} & \cdots & v_{nm} \end{array}\right]$$

(2) Determine the value of evaluation index entropy:

We compute the relative ratio of $v_{ij}$ for each evaluation object i as $p_{ij}$ , and the calculation formula is as follows:

(8) $$p_{ij}=\frac{v_{ij}}{\sum_{i=1}^{n}v_{ij}}$$

Then, the entropy $E_{j}$ of the $j^{th}$ evaluation index is defined as:

(9) $$E_{j}=-\frac{1}{\ln n}\overset{n}{\underset{i=1}{\sum }}p_{ij}\cdot \ln p_{ij}$$

A special case is that if $p_{ij}=0$ , then $p_{ij}\cdot \ln p_{ij}=0$ .

(3) Determine the weight of index:

The entropy weight $\omega_{j}$ of each evaluation index can be expressed as:

(10) $$\omega_{j}=\frac{1-E_{j}}{\sum_{j=1}^{m}\left(1-E_{j}\right)}$$

#### 3.3.3. Calculation of Connection Degree

If the evaluation sample is set X , then the *K*-element connection degree of $\left(X,B_{1}\right)$ can be defined as [44]:

(11) $$\begin{array}{ll} \mu_{X\sim B_{1}}= & \overset{n}{\underset{j=1}{\sum }}\omega_{j}\mu_{X_{t}\;∼B_{1}}\\ & =\overset{n}{\underset{j=1}{\sum }}\omega_{j}b_{j,0}I_{0}+\overset{n}{\underset{j=1}{\sum }}\omega_{j}b_{j,1}I_{1}+\overset{n}{\underset{j=1}{\sum }}\omega_{j}b_{j,2}I_{2}+\cdots +\overset{n}{\underset{j=1}{\sum }}\omega_{j}b_{j,K-2}I_{K-2}+\overset{n}{\underset{j=1}{\sum }}\omega_{j}b_{j,\;K-1}I_{K-1} \end{array}$$

If we let $f_{1}=\overset{n}{\underset{j=1}{\sum }}\omega_{j}b_{j,0},\;f_{2}=\overset{n}{\underset{j=1}{\sum }}\omega_{j}b_{j,1},\;\cdots ,f_{K-1}=\overset{n}{\underset{j=1}{\sum }}\omega_{j}b_{j,K-2},\;f_{K}=\overset{n}{\underset{j=1}{\sum }}\omega_{j}b_{j,K-1}$ , then Equation (11) can be transformed into:

(12) $$\mu_{X\sim B_{1}}=f_{1}I_{0}+f_{2}I_{1}+f_{3}I_{2}+\cdots +f_{K-1}I_{K-2}+f_{K}I_{K-1}$$

where $f_{K}$ represents the possibility that the evaluation sample belongs to the $K^{th}$ level of the standard. We computed a continuous measure of security rating μ , the connection degree, by setting $I_{i}=1-\frac{2i}{K-1}$ . As a result, we can rewrite Formula (12) as

(13) $$\mu_{X\sim B_{1}}=\overset{K}{\underset{i=1}{\sum }}f_{i}\left(1-\frac{2\left(i-1\right)}{K-1}\right)=f_{1}+f_{2}\left(1-\frac{2}{K-1}\right)+f_{3}\left(1-\frac{4}{K-1}\right)+\cdots +f_{K-1}\left(-1+\frac{2}{K-1}\right)-f_{K}$$

The connection degree is computed for each DPSIR component, for each year at each city. The connection degree is linearly transformed from [0, 1] to [1, 5] to be compared with other measures in Section 3.3.4.

#### 3.3.4. Measures of Water Resources Security

In this paper, we computed a discrete measure of overall water security rating (1 to 5) for each city and each year using the confidence level grading method [88]:

(14) $$\underset{k}{\min}h_{k}=f_{1}+f_{2}+\cdots +f_{k}>\lambda \;,\;\;\;\;\;\;\;\;\;\;\mathrm{where}\;\;\;k=1,2,\cdots ,K$$

Here, $h_{k}$ is the property measure, and λ is the confidence threshold. λ should not be too large, otherwise the evaluation results tend to be conservative and stable; λ should not be too small, otherwise the reliability of the results becomes poor, and the evaluation results tend to be over-positive. It is generally recommended that λ be taken in [0.5, 0.7] [58,59,89]. This threshold parameter λ is often subjectively chosen in this range of [0.5, 0.7] in the previous literature [38,39,40,41]. For a selected λ , the evaluation security rating is selected as the minimal *k* such that $h_{k}$ is greater than λ . For example, if $h_{1}=f_{1}<\lambda$ and $h_{2}=f_{1}+f_{2}>\lambda$ , then the evaluation security rating is *k* = 2.

**Figure 2:**
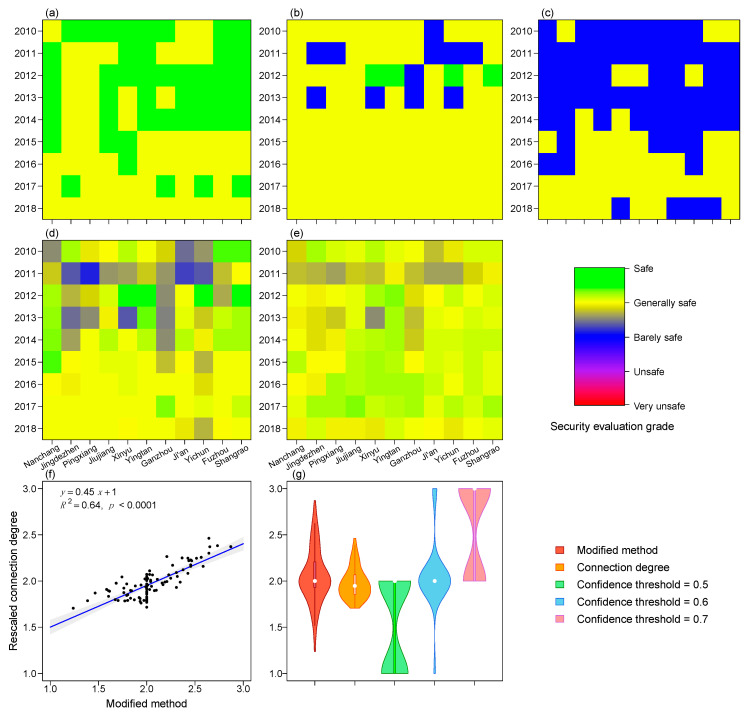
(**a**–**c**) Water resource security grade with λ = 0.5, 0.6, 0.7; (**d**) water resources security using the graded confidence threshold; (**e**) connection degree rescaled from 0–1 to 1–5; (**f**) correlation between the modified method and the connection degree; (**g**) distributions of water security scores in all methods; here, 1 is Safe, and 3 is Barely safe.

In order to avoid the subjectivity of the choice of λ , we proposed a simple modification of the confidence level grading method. We sampled 100 λs uniformly from the empirical range of [0.5, 0.7], and we repeated the calculations of the minimal *k* such that $h_{k}>\lambda$ for 100 times for each of the 100 different λs . Then, we averaged the results from these 100 λs to determine the grade of each index. This gave us a continuous measure of water resources security. We showed in Figure 2 that this measure correlates strongly with the connection degree.

### 3.4. Statistical Analysis

#### 3.4.1. Repeated Measures Correlation

Repeated measures correlation (rmcorr) was first introduced by Bland and Altman in biostatistics to analyze the correlations between paired repeated measures, which are two corresponding measures assessed for each individual/site on multiple occasions [90,91]. For repeated measures, the independence assumption adopted by simple correlation/regression is often violated. Repeated measure correlation addresses this non-independence among observations by using analysis of covariance (ANCOVA) to statistically adjust for inter-site variability. Conceptually, rmcorr is similar to a regression model that assumes a common slope and varying intercept for each group (in our case, city or year). By removing measured variance between sites, rmcorr provides the best-fit parallel regression lines with varying intercepts and the same slope. The rmcorr coefficient ranges from −1 to 1, similar to Pearson correlation [92].

In our study, we used rmcorr to analyze the correlations among pairs of DPSIR water security ratings (for example, between D and P) that were computed for each year and each city. We assessed the repeated correlations both longitudinally and cross-sectionally. In the longitudinal assessment, we used rmcorr to adjust for the variances across cities. In other words, we analyzed how a pair of factors (e.g., D and P) covaried over the years. In the cross-sectional assessment, we used rmcorr to adjust for the variances across years instead. In other words, we analyzed how a pair of factors (e.g., D and P) covaried across cities. Repeated measures correlation analysis was implemented in R programming language using the “rmcorr” package [92].

#### 3.4.2. Linear Mixed Models

In this study, Linear Mixed Models (LMMs) [93] were used to assess the temporal trend of the DPSIR factors over the years. In our case

$$f=\beta_{year}Y+\tau_{city}+\epsilon$$

Here, *f* stands for D, P, S, I, or R, and the LMMs analysis was applied for each factor separately. $\beta_{year}$ is the slope parameter that quantifies the temporal trend of each factor, Y is the year, $\tau_{city}$ is the city ID, and ϵ is the random error. We assume a fixed effect of Year and a random effect of City. The analysis was implemented in R programming language using the “lmer” function in package “lme4” [94].

## 4. Results

### 4.1. Water Resource Security Evaluation in Jiangxi Province Using the Modified Confidence Threshold Method

Using Fuzzy set pair analysis and the entropy weight method, we calculated the water resource security for each city and each year. In our dataset, the calculated weights for each DPSIR factor are shown in the table below (Table 3).

The water resource security evaluation method described in this paper depends critically on a confidence level parameter λ . It has been suggested in the literature that λ should be chosen from [0.5, 0.7]. However, the subjective selection of λ can have consequential impacts on the estimated water security. To illustrate the caveat of this subjectivity in the selection of λ , we compared five different measures of water resource security, three measures using subjective thresholds λ  = 0.5, 0.6, 0.7, the measure using our proposed modified method, and finally the connection degree measure. The estimated water resource security with λ=0.5, 0.6, 0.7 is shown in Figure 2a–c, respectively. A lower λ tends to give optimistic estimates that the water security is classified as Safe or Generally Safe for all cities and all years, whereas a higher λ tends to give pessimistic estimates that the water security is classified as Barely Safe for significant proportions of cities and years. Furthermore, this subjective selection of λ can lead to contradictory conclusions. When comparing the water resource security in 2010–2014 and 2015–2018, with λ=0.5 , most cities change from Safe to Generally Safe, showing a declining temporal trend in safety (Figure 2a). However, with λ=0.7 , most cities change from Barely Safe to Generally Safe, showing an increasing temporal trend in safety (Figure 2c). To avoid this subjectivity in parameter selection of λs , we used a modified method that utilizes a range of uniformly sampled λs (Figure 2d). We further compared this modified method and the connection degree. The connection degree is rescaled from 0–1 to 1–5 for equal comparison (Figure 2e). The modified method is strongly correlated with the connection degree (Figure 2f, R^2^ = 0.64, *p* < 0.001). The distributions of water security scores across cities and years in all methods (the modified method, the connection degree, and old methods with λ=0.5, 0.6, 0.7 are shown in Figure 2g. The score from the modified method has a shape that is closest to a normal distribution and lends itself to more convenient statistical tools.

Overall, we find that the water resource security of 11 cities in Jiangxi province is generally safe, ranging from Barely Safe to Safe (Figure 2d–e). We did not find Unsafe or Very Unsafe grades in any of the 11 cities from 2010–2018. In addition, we found that the water resource security changed from a more inconsistent and volatile grade (Barely Safe to Safe) before the year of 2014, to a more consistent and stable grade (Generally Safe) since the year of 2014. This change reflected the regional water resource managers’ efforts in water resources management. The overall degree of environmental remediation is strengthening over the years.

### 4.2. Temporal and Spatial Dynamics of Water Resource Security in Jiangxi Province

**Figure 3:**
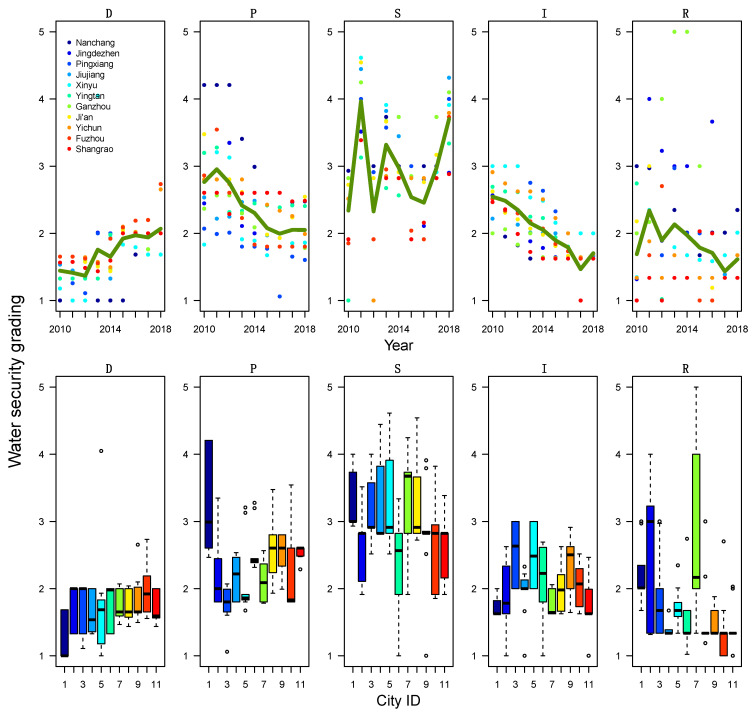
Time series and boxplot of water security grading of each city during the years 2010–2018.

We further investigated the DPSIR components of the water resources security and how they varied over the years and across different cities. Temporally, we showed the DPSIR scores of each city as a function of year (Figure 3, upper panel) to investigate the temporal trend. Spatially, we showed the variation of the DPSIR scores across years as a function of city (Figure 3, lower panel) to investigate the spatial differences in water resources management. Each color denotes a city, and the green line in the upper row showed the yearly averages of water security score across the eleven cities.

Linear Mixed Models (LMMs) were used in analysis to account for repeated measures and assess the statistically significance of the temporal trends of the DPSIR factors. Specially, we showed a significant descending trend (from 5-Unsafe to 1-Safe) in the Pressures, Impact, and Response factors. In addition, we showed a significantly ascending trend (from 1-Safe to 5-Unsafe) in the Driver factor. The State factor remains unchanged during the time period of the study (Table 4). The Pressures factor significantly improved over the years (*p* < 0.001). Considering the four water resources pressure indices used in this study, the decrease in the security level shows that the utilization rate of water resources in various industries is getting higher, and the pressure on water resources security is gradually weakened. The Impact factor significantly improved over the years (*p* < 0.001). The increasing in index I indicates that the urbanization level increases, and the greening rate and the water supply assurance rate of these cities are getting higher over the study period. The response factor also significantly improved over the years (*p* = 0.017), showing an increase in the laws and rules in favor of sustainable water usage. The safety of the Driver factors significantly declined over the years (*p* < 0.001). This indicates that the economic and industrial structure of the cities in Jiangxi Province rapidly developed but exposed more risks to water security over the study period. The State factor remained unchanged during the period of study (*p* > 0.05), indicating a sustainable water quality.

The variations of DPSIR factors over the years are computed for each city. We showed a significant difference among the cities. As the capital city of the province, the capital city Nanchang (City #1 in dark blue, Figure 3) has the most industry and economic growth among the 11 cities (the safest/lowest D factor); the heaviest pollution problem, land-use charges, and population growth (the least safe/highest P factor); and a relatively low water quality (a relatively unsafe/high S factor). On the other hand, Fuzhou (City #10 in red, Figure 3) is a city under-development with a high Driver factor rating, a low Pressures factor rating, and a low State factor rating, the opposite of Nanchang. This anti-correlations among factors are further explored in the next section. Moreover, we noticed that some cities went through more changes over the years compared to other cities. In particular, the cites Jingdezhen (City #2) and Ganzhou (City #7) have a significant higher variance in the Response factor compared to other cities, showing a significant impact of taxes and environmental laws on water management in these cities.

### 4.3. The Cross-Sectional and Longitudinal Inter-Correlations among the DPSIR Subsystems

**Figure 4:**
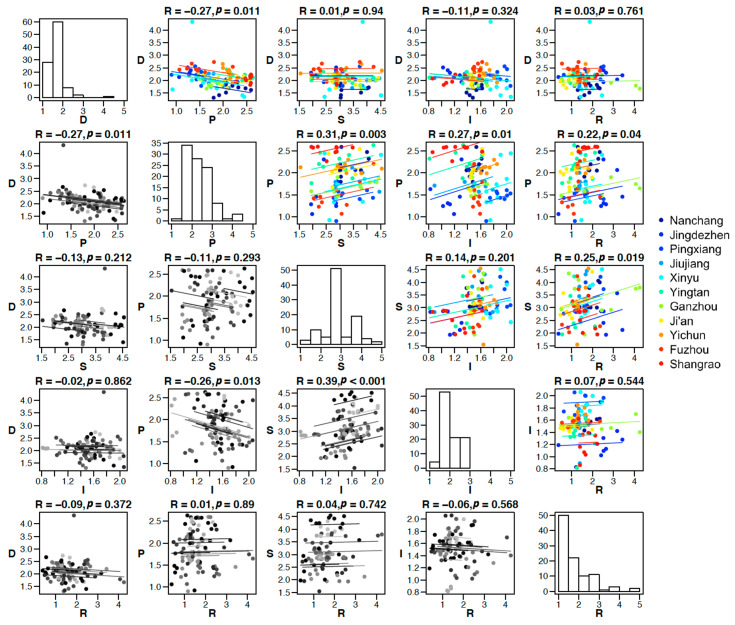
The repeated measures correlations among the DPSIR water security estimates of the 11 cities in Jiangxi Province during 2010–2018; data in the upper triangular are grouped by cities, and different colors indicate different cities; data in the lower triangular are grouped by years, and different intensity of the grey indicates different years.

In this paper, we assessed empirically the inter-connections among the 5 subsystems in the DPSIR framework, namely, Driver, Pressure, State, Impact, and Response. Temporal trends were removed for each factor, and the inter-correlations were computed based on residuals. In particular, we assessed the inter-correlations among the DPSIR components both cross-sectionally and longitudinally, by controlling the temporal (year) and spatial (city) factor, respectively, using repeated measures correlation. The diagonal panels in Figure 4 showed the histograms of the DPSIR security ratings across years and cities. The upper triangular of Figure 4 showed the repeated measures correlations among DPSIR factors over the years (city is treated as a random effect). Data points in these plots are grouped by cities (the color scheme is the same as in Figure 3); different colors here are different cities, and a separate regression line is drawn for each city. The lower triangular of Figure 4 showed the repeated measures correlations among DPSIR factors across cities (year is treated as a random effect). Data points in these plots are grouped by years, and the different intensity of the grey indicates different years (more recent years are represented by darker colors). A separate regression line was drawn for each year. The regression’s coefficient and associated p-values are shown in the title of each subplot (Figure 4).

Driver and Pressures factors are negatively correlated, both over the years (R = −0.27, *p* = 0.011) and across cities (R = −0.27, *p* = 0.011). This is in line with the DPSIR framework that an increase in Driver should lead to a decrease in the Pressures score (e.g., high industry development leads to more pollution). Increasing Drivers such as population and economic and social development promotes the development of the city but at the same time imposes pressures on water supply demand and sewage treatment, which would decrease the safety level in Pressure factors. Pressure and States correlate over the years (R = 0.31, *p* = 0.003). Higher Pressure would lead to Poorer State (e.g., lower water quantity and quality). State factor does not correlate significantly with Impact factor over the years (*p* > 0.05) but correlates significantly with Impact factor across cities (R = 0.39, *p* < 0.001). Pressure and Impact positively correlate over the years (R = 0.27, *p* = 0.01) and negatively correlate across cities (R = −0.26, *p* = 0.013). Over the years, increasing water demand due to agricultural and residential water use leads to a decrease in the safety rating of the Impact of water security (e.g., decreasing greening coverage). Across the cities, however, cities with high Pressure safety have low Impact safety. Finally, the Response factor correlated significantly with both Pressure (R = 0.22, *p* = 0.04) and State (R = 0.25, *p* = 0.019) over the years. Good responses to water threats can indeed decrease water pressure and lead to better water/soil states. Impact factor did not significantly react to responses in the study region (*p* > 0.05).

## 5. Discussion

In this paper, we used the DPSIR framework to evaluate regional water resources security in Jiangxi Province in China. Our study constructed a water resources security evaluation index system and evaluation grade standards for 11 cities in Jiangxi Province. Fuzzy set pair analysis method and entropy weight method were used to evaluate water security through a modified confidence-threshold method. We analyzed the temporal trend and spatial variations of water resources security, separately for the Driver, Pressure, State, Impact, and Response subsystems. In addition, we empirically assessed the inter-correlations among the five subsystems of the DPSIR system, both longitudinally and cross-sectionally by controlling for city and year respectively. We revealed a more complicated and region-specific DPSIR flow and suggested that such region-specific networks are informational in guiding local policy making.

The evaluation of water resources security in the DPSIR framework depends critically on a confidence threshold parameter λ . This threshold λ was usually subjectively chosen in the literature. The subjective choice of λ varied from study to study, some of the choices are 0.55 [39], 0.6 [40], and 0.7 [41]. In our paper, we showed that this subjectivity would lead to inconsistent and even opposite conclusions in water resources evaluation and is prone to errors (Figure 2). To address this caveat, we proposed a simple modification that averages water security estimates over a range of λs . In this method, researchers no longer had to specify a single subjective λ value. We compared our modified method with connection degree and found that these two measures correlate strongly with each other. We recommend future researchers to avoid using subjective λs and use alternative continuous measures such as our modified method or the connection degree.

We comprehensively evaluated Jiangxi Province’s water resources security of all cities from 2010 to 2018. We can see that since 2014, Jiangxi Province’s water resources become more stable and consistent across cities and mostly fall in the category of “Generally Safe”. Through trend analysis, we were able to identify differential temporal trends among the DPSIR factors. The water resources security in Jiangxi Province has been improving over the years in many aspects. The water security levels of the indices pressure (P), impact (I), and Response (R) have decreasing trends over time and are becoming safer over the years. This is related to the continued construction of water-saving reconstruction projects in Jiang provinces in recent years. Moreover, the development and utilization rate of water resources in Jiangxi Province is increasing, which also makes the water resources security safer over the years. The index driver (D) has an increasing trend and is becoming less safe, and more attention is needed in regulating the Driver. This manifests in a number of factors including the increase of population density and the decrease of GDP growth rate. Jiangxi Province has attracted a large number of migrant populations in recent years, resulting in population aggregation, which in turn results in a relative increase of domestic water consumption of urban residents and a relative reduction of per capita water and economic resources. The state (S) remains unchanged.

Despite the general temporal trend, we identified significant differences among cities. The water resources security in some cities are relatively poorer than others and require more attention. Cities like Nanchang, Ganzhou, and Jiujiang are developing rapidly socio-economically; the GDP growth rates are large; these cities not only attract a large number of foreign populations, but also have local population aggregation, resulting in the relative increase of domestic water consumption of urban residents. The per capita water resources are relatively reduced, and the contradiction between supply and demand of water resources become prominent. However, cities like Yingtan, Shangrao and Fuzhou are rich in annual rainfall and per capita water resources. The domestic water consumption of urban residents and the water consumption of 10,000-yuan industrial added value are less, and the reuse rate of industrial water and sewage treatment rate are also higher than the average value in the province.

**Figure 5:**
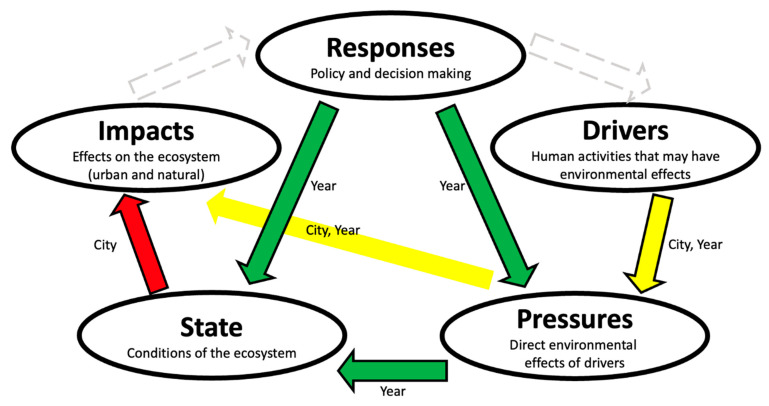
A causal network of DPSIR model used for water resources security gradings in this study. The green arrows represent correlations over years, the red arrows represent correlations across cities, and the yellow arrows represent correlations both over years and across cities. The dashed arrows are links in the DPSIR framework that did not show empirical correlations in our study region.

Furthermore, we empirically assessed the inter-correlations among the DPSIR subsystems. We argue that despite the general causal flow in the DPSIR framework, the sensitivities of changes between different subsystems should vary from region to region. It is important to assess the region-specific intercorrelations among the DPSIR subsystems empirically. In this paper, we tested such correlations both longitudinally and cross-sectionally using repeated measures correlations. Longitudinal correlations reflect more the intrinsic associations between the rise and fall of different pairs of factors, whereas cross-sectional correlations reflect more the regional heterogeneity in water resources management. The results are summarized in Figure 5. The general DPSIR framework has a forward loop in the order of D-P-S-I-R, and feedback edges (R, D), (R, P) and (R, S) represent the Responses in response to Drivers, Pressures, and States. In our study region, there are informational deviations from this standard framework in inter-correlations among the DPSIR subsystems. For longitudinal correlations, Impact seems to be detached from the rest of the 4 subsystems. In particular, Responses do not seem to arise from Impacts but rather from Pressures and State directly. In addition, the fact that Responses correlate with Pressure and State but not Driver, indicate that the local policies are less targeted at regulating Driver compared to managing Pressures and State. Our analysis suggested that future policies should consider regulating Driver. We suggest that relevant departments can increase investment in economic development, accelerate the development of advanced water resources technology, tap the exploitable potential of water resources, and resolve the problem of increasing demand for water resources from the source. Moreover, it is beneficial to formulate effective water-saving strategic plans, promote the recycling of water resources, and improve the optimal allocation of water resources. For cross-sectional correlations, we observed significant correlations between (S, I) and (P, I). This suggests the regional heterogeneity in the causal influences on the Impact factor. We think that these region-specific interconnections and their deviances from the standard DPSIR framework carry useful information about the local water resources system and can assist local governors in making better local water resources policies. Our approach of empirical assessment of the region-specific connectivity in various subsystems over space and time can serve as a reference for future regional water resources evaluation studies.

## 6. Conclusions

This study used the DPSIR framework and assessed the temporal and spatial dynamics of water resources security in Jiangxi Province, China. In this study, we demonstrated that methods using the subjective confidence threshold in assessing water resources security can lead to contradictory conclusions depending on the choice of the confidence threshold parameter. To address this limitation, we proposed a modified method that samples a range of threshold values and pools them to obtain more objective measures of water resources security.

From the analysis of the temporal trend and spatial variation of DPSIR-based water security level in Jiangxi Province, we found that while Pressures, Impacts, and Responses are becoming safer over the years, the Driver factor is becoming less safe and requires more attention. The increase in population density and the decrease in per capita water resources have made the contradiction between supply and demand of water resources more prominent in Jiangxi Province.

Through longitudinal and cross-sectional correlational analysis among the DPSIR subsystems in Jiangxi Province, we found regional deviances of inter-correlations among the DPSIR nodes from the general DPSIR causal chain framework. In particular, the Responses in this area correlate with Pressure and State but not with Driver. This suggests that local government should direct future policies towards improving the security level of Driver, and relevant departments should increase the development of water resources potential, formulate water-saving strategic measures, and optimize the allocation of water resources.

## Figures and Tables

**Table 1:** Social economic factors used in the DPSIR system. Here, index type “+” indicates that higher values are more desirable for the particular factor whereas “-” indicates that lower values are more desirable.

Subsystem	Factors	Unit	Calculation	Meaning of Index	Index Type	References
Driver	Per capita GDP (D1)	yuan/person	GDP/population	It indicates the driver of economic development on water resources security	+	[47,48,71,72]
	Population density (D2)	person/km^2^	Total population/land area	It indicates the driver of population density on water security	-	[47,60,72,73]
	Urbanization rate (D3)	%	Non-agricultural population/total population	It indicates the driver of regional development on water resources security	+	[15,47,48,60]
	Annual GDP growth rate (D4)	%	Statistical data	It indicates the driver of economic development intensity to water resources security	+	[15,47,60]
Pressure	Water use for each 10,000 yuan of GDP (P1)	m^3^/10,000 yuan	Total amount of water use/total GDP	It indicates the pressure of economic development intensity on the quantity of water resources	-	[47,60,71,72]
	Wastewater discharge for each 10,000 yuan of GDP (P2)	m^3^/10,000 yuan	Wastewater discharge/total GDP	It indicates the pressure of industrial development on the quality of water resources	-	[47,60]
	Water use for each 10,000 yuan of agricultural output (P3)	m^3^/10,000 yuan	Total amount of irrigated water use/total output value of farming	It indicates the pressure of agricultural development on the quality of water resources	-	[47,60,71]
	Per capita daily consumption of tap water for residential use (P4)	L/day	Statistical data	It indicates the pressure of human life on the quantity of water resources	-	[47,60,73,74]
State	Per capita water resource quantity (S1)	m^3^/person	Total amount of water resources/total population	It indicates the per capita state of water resources	+	[15,23,47,60,71,74,75,76,77]
	Per unit area water resource quantity (S2)	10,000 m^3^/km^2^	Total amount of water resources/land area	It indicates the per unit area water resource state.	+	[23,72]
Impact	Energy consumption for each 10,000 yuan of GDP (I1)	Tons of SCE /10,000 yuan	Total Energy Composition/total GDP	It indicates the Potential impact of resource utilization on water resources	-	[74]
	Rate of green coverage area to developed area (I2)	%	Statistical data	It indicates the response of surface water storage to water resources	+	[60,71,74]
	Proportion of tertiary industry in GDP (I3)	%	Statistical data	It indicates the impact of water resources system on industrial structure	+	[47,60]
Response	Utilization rate of water resources (R1)	%	Total amount of water use/total amount of water resources	It indicates the response of water resources quantity security	-	[15,47,72]
	Urban sewage treatment rate (R2)	%	Statistical data	It indicates the response of standard discharge of sewage	+	[60,71,74,75,76]

GDP: gross domestic product; SCE: standard coal equivalent.

**Table 2:** Grades of water resource security evaluation.

Factor Level	Index Level	Index Type	1—Safe	2—Generally Safe	3—Barely Safe	4—Unsafe	5—Very Unsafe
Driver (D)	D1 (yuan)	+	>75,000	55,000–75,000	35,000–55,000	15,000–35,000	<15,000
	D2 (person/km^2^)	-	<250	250–2000	2000–3750	3750–5500	>5500
	D3 (%)	+	>70	50–70	30–50	10–30	<10
	D4 (%)	+	>10	8–10	5–8	3–5	<3
Pressure (P)	P1 (m^3^)	-	<300	300–600	600–1000	1000–1500	>1500
	P2 (m^3^)	-	<20	20–30	30–40	40–60	>60
	P3 (m^3^)	-	<500	500–1000	1000–1500	1500–2000	>2000
	P4 (L/day)	-	<70	70–120	120–170	170–220	>220
State (S)	S1 (m^3^)	+	>3000	1700–3000	1000–1700	500–1000	<500
	S2 (10,000 m^3^/km^2^)	+	>200	200–150	150–100	100–50	<50
Impact (I)	I1 (Tons of SCE)	-	<0.5	0.5–1	1–2	2–5	>5
	I2 (%)	+	>40	30–40	20–30	10–20	<10
	I3 (%)	+	>60	40–60	20–40	5–20	<5
Response(R)	R1 (%)	-	<5	5–15	15–30	30–45	>45
	R2 (%)	+	>90	80–90	70–80	60–70	<60

**Table 3:** Weights of each DPSIR factor.

D1	D2	D3	D4	P1	P2	P3	P4	S1	S2	I1	I2	I3	R1	R2
0.060	0.067	0.065	0.150	0.052	0.051	0.049	0.040	0.075	0.070	0.045	0.062	0.079	0.050	0.086

**Table 4:** LMM results of temporal trend analysis of the DPISR water resources security.

	D		P		S		I		R	
Predictors	Estimates	*p*	Estimates	*p*	Estimates	*p*	Estimates	*p*	Estimates	*p*
(Intercept)	1.27	**<0.001**	2.99	**<0.001**	2.79	**<0.001**	2.70	**<0.001**	2.15	**<0.001**
	(0.87–1.67)		(2.58–3.40)		(2.39–3.20)		(2.30–3.10)		(1.74–2.57)	
years	0.09	**<0.001**	−0.12	**<0.001**	0.03	0.212	−0.13	**<0.001**	−0.06	**0.017**
	(0.04–0.14)		(−0.17–−0.07)		(−0.02–0.08)		(−0.18–−0.08)		(−0.11–−0.01)	
Random Effects										
$\sigma^{2}$	0.11		0.11		0.45		0.04		0.43	
ICC	0.14		0.51		0.16		0.62		0.32	
N	11citys		11citys		11citys		11citys		11citys	
Observations	99		99		99		99		99	
Marginal R^2^/ Conditional R^2^	0.299/0.397		0.304/0.660		0.013/0.172		0.518/0.815		0.040/0.346	

ICC: Intraclass correlation coefficient. The significant *p* values are bolded.

## Data Availability

Publicly available datasets were analyzed in this study. This data can be found here: https://data.cnki.net/NewHome/index, accessed on 20 November 2020 and http://data.stats.gov.cn/, accessed on 20 November 2020.
