# Correction: Lu et al. An Assessment of Temporal and Spatial Dynamics of Regional Water Resources Security in the DPSIR Framework in Jiangxi Province, China. *Int. J. Environ. Res. Public Health* 2022, *19*, 3650

**DOI:** 10.3390/ijerph22040630

**Published:** 2025-04-17

**Authors:** Mengtian Lu, Xiaoying Wang, Weihong Liao, Chao Wang, Xiaohui Lei, Hao Wang

**Affiliations:** 1 Institute of Municipal Engineering, College of Civil Engineering and Architecture, Zhejiang University, Hangzhou 310030, China; 11512052@zju.edu.cn (M.L.); wanghao@iwhr.com (H.W.); 2 Anhui Water Conservancy Technical College, Hefei 231603, China; wxy769662707@163.com; 3 Key Laboratory of Simulation and Regulation of Water Cycle in River Basin, China Institute of Water Resources and Hydropower Research, Beijing 100038, China; wangchao@iwhr.com (C.W.); lxh@iwhr.com (X.L.)

Siyu Wang was included as an author in the original publication [1] and has been removed. The affiliations and affiliation numbers have been adjusted accordingly. The corrected Author Contributions statement appears here.

**Author Contributions:** Conceptualization, M.L., X.W. and W.L.; methodology, M.L.; formal analysis, M.L. and C.W.; writing—original draft preparation, M.L.; writing—review and editing, M.L., X.W. and W.L.; visualization, M.L.; supervision, W.L., X.L. and H.W. All authors have read and agreed to the published version of the manuscript.

The authors state that the scientific conclusions are unaffected. This correction was approved by the Academic Editor. The original publication has also been updated.
